# A Self-Powered Biosensor for the Detection of Glutathione

**DOI:** 10.3390/bios10090114

**Published:** 2020-09-03

**Authors:** Brandon G. Roy, Julia L. Rutherford, Anna E. Weaver, Kevin Beaver, Michelle Rasmussen

**Affiliations:** Department of Chemistry, Lebanon Valley College, Annville, PA 17003, USA; br005@lvc.edu (B.G.R.); jr012@lvc.edu (J.L.R.); aew006@lvc.edu (A.E.W.); kjb006@lvc.edu (K.B.)

**Keywords:** glutathione, neurodegenerative disorder, self-powered, biofuel cell, biosensor, bilirubin oxidase

## Abstract

Glutathione is an important biological molecule which can be an indicator of numerous diseases. A method for self-powered detection of glutathione levels in solution has been developed using an enzymatic biofuel cell. The device consists of a glucose oxidase anode and a bilirubin oxidase cathode. For the detection of glutathione, the inhibition of bilirubin oxidase leads to a measurable decrease in current and power output. The reported method has a detection limit of 0.043 mM and a linear range up to 1.7 mM. Being able to detect a range of concentrations can be useful in evaluating a patient’s health. This method has the potential to be implemented as a quick, low-cost alternative to previously reported methods.

## 1. Introduction

Glutathione (GSH) is a tripeptide produced naturally within all domains of life. Over 130 years of research have been focused on this chemical compound, and many molecular processes involving GSH have been discovered to aid in the detoxification and regulation of cell life at all levels [1]. Glutathione is a common biomarker that can be useful in the detection and diagnosis of many diseases [2]. While no disease can be diagnosed using glutathione concentrations alone, it can still be used as an indicator of possible health issues. When the levels of GSH are significantly higher, it has been shown that the chances of a patient being diagnosed with a neurodegenerative disease increase [3]. Being able to detect and quantify GSH in biological samples accurately is beneficial as cases of these disorders increase. There is also the potential for diagnostic measures over time to determine the progression of diseases like Parkinson’s or Alzheimer’s [3].

Fluorimetric and colorimetric methods are the most common for detecting glutathione, often using nanoparticles or quantum dots [4,5,6]. An interesting recent paper by Chu et al. describes the use of a smartphone platform for fluorescent detection of glutathione in human serum [7]. Their device uses two fluorescent probes, carbon dots, and Au nanoparticles, which produce two colors, blue and orange. The Au nanoparticle fluorescence is quenched with copper ions, but the fluorescence is restored in the presence of glutathione. The ratio of the fluorescence is measured using a color-recognizing app on a smartphone application. While these reported methods have the potential to make very sensitive measurements, they require highly controlled probe concentration, complex solution preparation, and a dark environment (for fluorescence). They also require expensive instrumentation and technical training which limits use in some locations. A portable method that removes these requirements would be advantageous for resource-limited areas. By using a self-powered enzymatic biosensor, several of these limitations can be addressed.

Self-powered is a classification of sensors first used by Katz et al. in 2001 to describe the determination of analyte concentration by measuring the power or current output of a fuel cell [8]. This technique had been used for earlier microbial fuel cells prior to the use of this classification. A self-powered device can replace large, expensive equipment and reduce the need for highly technical training. Self-powered sensors are essentially fuel cells where the analyte is detected by either an increase or decrease of current and power output. This change in current and power is due to either activation or inhibition at one of the electrodes (either anode or cathode depending on the analyte). This type of sensor has been reported for several analytes, including lactate [8], nitroaromatic explosives [9], EDTA [10], arsenic [11], acetaldehyde [12], and herbicides [13].

**Figure 1:**
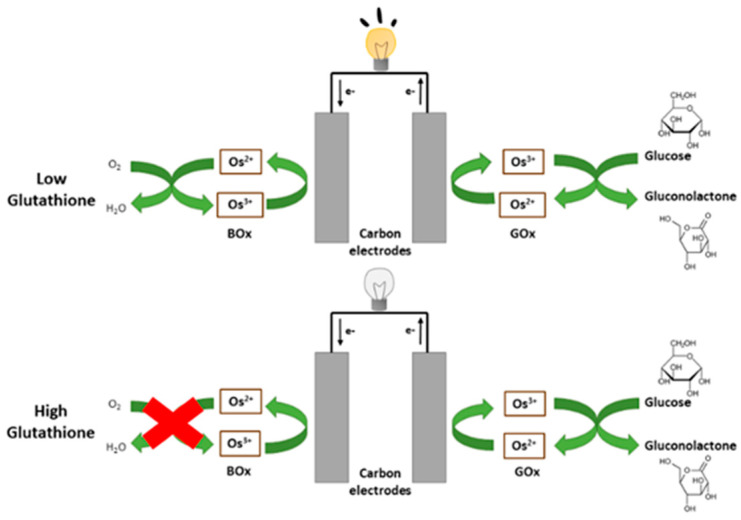
Schematic of a self-powered enzymatic biosensor for the detection of glutathione.

Glutathione has been shown to inhibit laccase, a commonly-used multicopper oxidase for oxygen reduction [14]. We chose to investigate laccase along with two other copper-containing oxygen reduction enzymes, bilirubin oxidase (BOx) and tyrosinase, for use in a glutathione sensor. In this paper, we confirmed this inhibition and incorporated BOx into an oxygen reduction electrode capable of detecting GSH. We combined this electrode with a glucose oxidase (GOx) anode to make a self-powered system to detect the concentration of glutathione in solution. As seen in Figure 1, the biosensor is a biofuel cell that uses glucose and oxygen as its fuels. The current produced by the device decreases with increasing amounts of glutathione due to inhibition at the biocathode making it possible to determine glutathione concentration.

## 2. Materials and Methods

### 2.1. Materials

2,2′-azino-bis(3-ethylbenzothiazoline-6-sulfonic acid (ABTS) diammonium salt, 2′-Azobis(2-methylpropionitrile) (AIBN), calf serum bovine donor, diethyl ether, D-glucose, glucose oxidase from *Aspergillus niger*, l-glutathione reduced, laccase from *Trametes versicolor*, and poly(ethylene glycol) diglycidyl ether (PEGDGE, Mn 500) were used as received from Sigma-Aldrich (St. Louis, MO, USA). 1-vinylimidazole was used as received from TCI America. Bilirubin oxidase was kindly provided by Amano Enzyme (Elgin, IL, USA). Tyrosinase from mushrooms was purchased from Calzyme Laboratories Inc. (San Luis Obispo, CA, USA). Acetone, ethanol, and methanol were purchased from Pharmco. Citric acid, potassium phosphate dibasic, and potassium phosphate monobasic were used as received from Fisher Scientific.

### 2.2. Electrode Fabrication

The osmium redox polymer used for all enzyme electrodes was prepared using the method reported by VandeZande et al. [15]. First, poly(1-vinylimidazole) (PVI) was synthesized by heating 6 mL of 1-vinylimidazole to 70 °C under N_2_. AIBN (0.5 g) was added and the reaction was run for 2 h. After cooling to room temperature, precipitate was dissolved in methanol and precipitated with acetone. Os(bpy)_2_Cl_2_ was synthesized using the procedure by Forster [16] with one crucial adjustment, and all steps were performed under N_2_. The osmium complexes were attached to the PVI by first dissolving 40 mg Os(bpy)_2_Cl_2_ in 30 mL ethanol. The solution was refluxed for 30 min and then PVI, 70 mg in 10 mL ethanol, was added. The mixture was refluxed for 72 h. After cooling, the osmium redox polymer was precipitated with diethyl ether.

Enzyme electrodes were prepared using the following procedure. Enzyme (13 mg/mL, 10 µL), PEGDGE (2.5 mg/mL, 18 uL), and PVI-Os(bpy)2 (10 mg/mL, 72 µL) were vortexed to mix. Then 10 µL aliquots were applied to glassy carbon electrodes (dia. 3 mm) and the electrodes dried overnight at ambient conditions. This same procedure was used for all enzyme electrodes with the only difference being the enzyme used. The crosslinker (PEGDGE) is able to react with free amine groups on the polymer as well as lysine groups on the enzymes. The resulting mesh-like network forms a layer on the electrode surface that swells in the presence of water, allowing for diffusion of substrate [17].

### 2.3. Electrochemical Experiments

All electrochemical measurements were taken on a CHI 660E series potentiostat. For cyclic voltammetry and amperometry experiments, a saturated calomel reference electrode and platinum counter electrode were used. The enzyme electrode was placed in 10 mL of a 0.1 M phosphate buffer solution (pH 7.4) for BOx or tyrosinase while 0.1 M citrate buffer (pH 4) was used for laccase. Stock glutathione solutions were prepared daily at a concentration of 1 or 10 mM, depending on the experiment. For self-powered sensor experiments, the electrode was placed in a 50 mM glucose/0.1 M phosphate buffer (pH 7.4), and aliquots of 10 mM glutathione solution were injected into the cell. For serum tests, the buffer was replaced with bovine serum with 50 mM glucose and aliquots of glutathione were injected into the cell. Current and power densities were calculated using the geometric area (0.071 cm^2^) of the cathode. All experiments were performed in triplicate, except the self-powered sensor tests which were replicated nine times. The plotted data show the averages of the measurements with the standard deviation used as the error.

## 3. Results and Discussion

### 3.1. Confirmation of Bioelectrocatalytic Response to Glutathione

**Figure 2:**
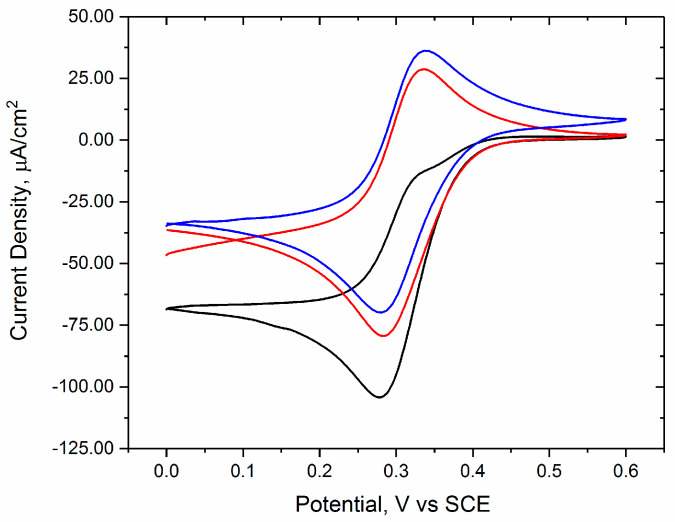
Representative cyclic voltammetry of bilirubin oxidase (BOx)/PVI-Os polymer electrodes in pH 7.4 0.1 M phosphate buffer in air (black line), N_2_ (red line), and air with glutathione (blue line). Data was collected at 10 mV/s with a platinum counter electrode.

To evaluate the possible inhibition of multicopper oxidases by glutathione, we immobilized each enzyme (BOx, laccase, and tyrosinase) using the same method. An osmium redox polymer was used for mediated electron transfer from the electrode to the enzyme. A crosslinker was used to bind the enzyme and polymer to form a film on the electrode. In Figure 2, the cyclic voltammetry data for three different conditions with BOx electrodes is shown. When the solution is purged of O_2_, the CV shows only the redox reactions of the osmium sites on the polymer (red line). When O_2_ is present (black line), oxygen reduction occurs, as shown by the increased reduction current below 0.4 V vs. SCE and the disappearance of the osmium oxidation peak. In the presence of glutathione and O_2_, BOx is inhibited, and the reduction current decreases significantly (blue line). From this data, it can be concluded that BOx electrodes can be used for inhibition-based detection of glutathione.

**Figure 3:**
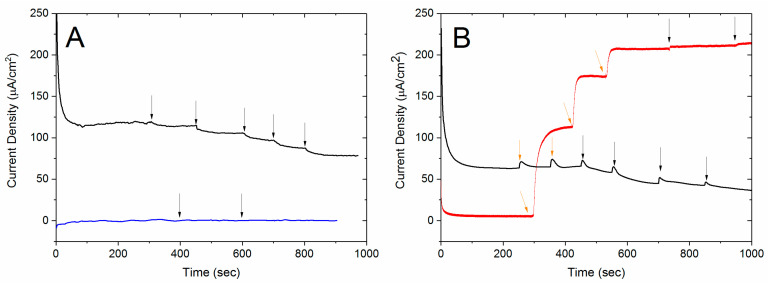
Amperometric data for four different conditions. The data was collected at 0.25 V vs. SCE in pH 7.4 0.1 M phosophate buffer with a platinum counter electrode. Black arrows indicate injections of glutathione (0.1 M per injection) and orange arrows indicate injections of glucose (25 mM per injection). (**A**) The black line shows data for a BOx electrode and the blue line indicates a BSA coated electrode. (**B**) The black line shows data for a BOx electrode, and the red line shows the response of a glucose oxidase (GOx) electrode.

**Figure 4:**
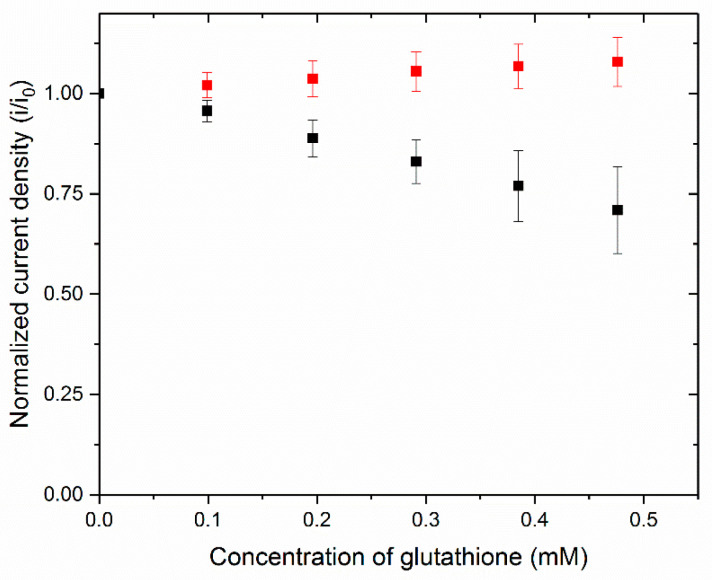
Normalized current density changes at varying glutathione concentrations in 0.1 M phosphate buffer, pH 7.4. The average data for BOx modified electrodes is seen in black and BSA coated electrodes in red.

Because there are several oxygen reduction enzymes that are inhibited by glutathione, we evaluated three types of electrodes to determine which would give the highest sensitivity to glutathione. Figure 3 shows the amperometric response at 0.25 V vs. SCE at increasing concentrations of glutathione for BOx electrodes in 0.1 M phosphate buffer (pH 7.4). When the BOx was replaced with BSA, the electrode no longer showed a response to the addition of glutathione. The black line in both plots is representative of data for a BOx electrode. The current density decreases with increasing glutathione concentration and shows no response to glucose, the substrate for the anode in the self-powered sensor. When BOx is replaced with BSA, the current density is significantly smaller and shows no response to glutathione (blue line in Figure 3A). The current was allowed to stabilize after each injection, and the current was recorded. The current data was normalized by dividing the current at each concentration by the initial current. The normalized results for three BOx electrodes were averaged and are shown in Figure 4 (black data points). As expected, the current density decreases as the concentration of glutathione increases. Average data for three BSA-modified electrodes (blue data points) is also included to show that no change in current is observed without the BOx on the electrode. Amperometric experiments were repeated with electrodes modified with either laccase or tyrosinase to determine which enzyme electrodes were most sensitive to glutathione (data not shown). The slopes of the standard curves (i.e., the sensitivity) are reported in Table 1. Laccase had the highest sensitivity with bilirubin oxidase slightly lower and tyrosinase with a significantly lower value. The major disadvantage to using laccase is that it works best in lower pH solutions which would require a membrane divider in our biofuel cell sensor. Because the BOx had good sensitivity and could operate at neutral pH, we chose to go forward with it for the cathode.

To use a GOx electrode for the anode, we first had to confirm that it is not affected by the presence of glutathione. Amperometric data for a GOx electrode is shown as the red line in Figure 3B. As expected, the current density increases with glucose concentration and shows no response to additions of glutathione. The concentration range of glucose in serum is 5–10 mM which is in the linear region of our glucose electrode. However, as can be seen in Figure 3B, the current produced by this electrode is significantly higher (at least one order of magnitude) so the current produced by the biofuel cell is limited by the cathode. This indicates that when a GOx electrode and BOx electrode are used, any change in current output from the biofuel cell as glutathione concentration changes is due to inhibition of the BOx cathode.

It is important that the biofuel cell not be sensitive to changes in glucose concentration. To verify this, we operated the biofuel cell at an applied voltage of 0 V at three glucose concentrations. First, we measured the current output in 5 mM glucose containing 100 µM glutathione. This is the expected lower limit for glucose concentration in serum samples. We injected aliquots of glucose to increase the concentration to 7.5 and 10 mM glucose (the upper limit for serum glucose concentration) while maintaining the glutathione concentration. As shown in Figure S1, after an initial increase due to the mixing of solution by the injection, the current stabilized at a similar value for all three glucose concentrations. Another concern is the high concentration of chloride found in serum, up to 0.1 M, which can inhibit bilirubin oxidase in some environments [18]. We performed a similar experiment with the biofuel cell in 10 mM glucose with 100 µM glutathione. After allowing the current to stabilize at 0 V, we injected solution containing potassium chloride while maintaining the glucose and glutathione concentrations. As shown in Figure S2, similar to the glucose tests, after an initial increase due to mixing, the current stabilized to a value similar to before the addition of chloride.

### 3.2. Testing the Complete Biosensor

**Figure 5:**
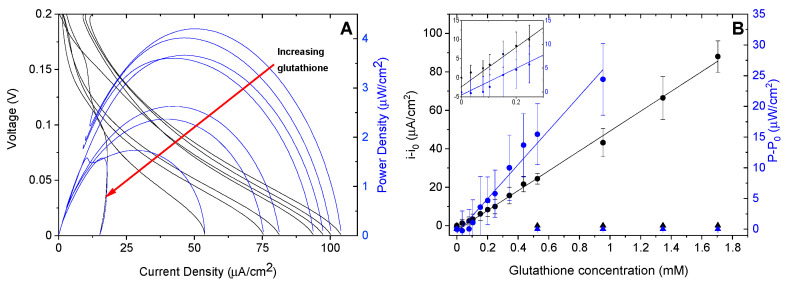
(**A**) Representative linear polarization (black lines) and power curves (blue lines) for the complete GOx/BOx self-powered sensor with increasing amounts of glutathione. (**B**) Background subtracted current (black circles) and power (blue circles) of GOx/BOx biofuel cell with increasing glutathione concentration. Data for control fuel cells with BSA cathodes is also included (triangles). The inset shows a closer view of the data for lower glutathione concentrations.

Figure 5A displays the linear polarization and power curves for the GOx anode and BOx cathode at increasing concentrations of glutathione. As the concentration of glutathione increases in the solution, the maximum current and maximum power decrease. The maximum current density and power were determined for nine sets of electrodes (a new anode and cathode for each trial). The data for each biofuel cell was normalized by subtracting from the current or power when no glutathione was present (i.e., the data was background subtracted using the initial current or power with no glutathione). This accounts for variations in the current production for individual fuel cells. The average was then calculated at each concentration and the averaged data is shown in Figure 5B. Linear fits for the data gave sensitivities of 51.6 ± 0.9 µA cm^−2^ mM^−1^ and 27.9 ± 1.5 µW cm^−2^ mM^−1^ for the current and power, respectively. The maximum power (P-P_0_) showed to an initial decrease in response which results in a less accurate linear fit (R^2^ value = 0.971). Therefore the limit of detection (LOD) was calculated using the error in the intercept of the linear fit to the maximum current data (i-i_0_) shown in Figure 5B (R^2^ = 0.996). The intercept was determined to be −2.39 ± 0.88 µA cm^−2^ which gives a LOD of 51 µM. This LOD is higher than some previously reported glutathione sensors, with some as low as 0.19 µM [4,7]. However, this is significantly lower than glutathione concentrations found in human serum and the linear range tends to be lower so samples must be diluted for those sensors. The linear range of our sensor is appropriate for determining glutathione in serum which has been shown to be ~80 µM in healthy patients [19]. This sensor makes it possible to determine concentrations both above and below the typical amount.

### 3.3. Application of the Biosensor to Serum Samples

**Figure 6:**
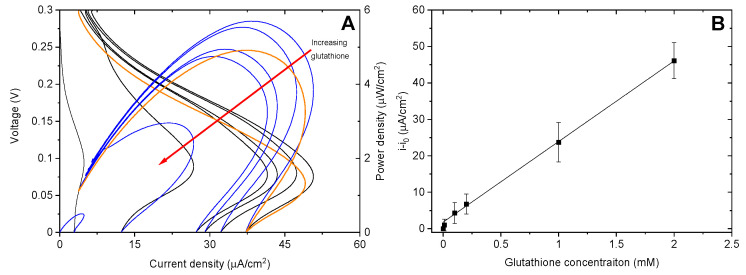
(**A**) Representative linear polarization (black lines) and power curves (blue lines) for a complete GOx/BOx self-powered sensor with increasing amounts of glutathione. (**B**) Background subtracted current densities of GOx/BOx biofuel cell with increasing glutathione concentration.

To verify that the biosensor can operate in real samples, the biofuel tests were repeated in bovine serum doped with glutathione. A representative set of polarization and power curves are shown in Figure 6A. In serum, the shape of both the polarization and power curves indicates decreased mass transport, resulting in lower currents close to 0 V. This is to be expected due to the increased viscosity of serum compared to aqueous solutions. A standard curve was generated from the average background subtracted maximum current densities (i-i_0_) of four biofuel cells (Figure 6B). Over the same concentration range for the buffer tests, the sensor shows a linear response to glutathione with a sensitivity of 22.1 ± 0.3 µA cm^−2^ Mm^−1^ and a limit of detection of 43 µM. This linear range is appropriate for determining glutathione in serum which has been shown to be ~80 µM in healthy patients [19]. This sensor makes it possible to determine concentrations both above and below the typical amount. The reversibility of the sensor was examined by collecting a new polarization curve in undoped serum after exposure to the highest gluathione concentration (2 mM). As can be seen in the bold orange lines in Figure 6A, the maximum power does not return to the initial value, however, the maximum current is almost completely restored. For four trials, the percent of signal restored after exposure to 2 mM glutathione was slightly higher than before exposure (102 ± 5%) indicating that the inhibition is reversible.

## 4. Conclusions

A self-powered method for the detection of glutathione using a biofuel cell was successfully developed and tested. Bilirubin oxidase was shown to be inhibited by glutathione. While the sensitivity of BOx to glutathione was not as high as laccase, its ability to operate at neutral pH removed the need for a membrane separator, simplifying the device. The use of bilirubin oxidase on the cathode and glucose oxidase on the anode showed a linear response to the change in concentration of glutathione in serum samples over the range of 43 to 2.0 mM. This method removes the requirement for highly controlled conditions and requires no large, expensive instrumentation.

The mechanism of inhibition by glutathione is not currently known. Preliminary results indicate noncompetitive inhibition but future work is aimed at elucidating the mechanism as well as testing the sensor with other reducing amino acids to evaluate the selectivity. Future work will also be aimed at studying the stability of the sensor. Preliminary results show a constant current output for the first two hours with a decrease in current output of ~20% after 12 h. The results indicate that the sensor is stable over the time needed to test samples, but more experiments are needed to prove this. Finally, to minimize the effects of decreased mass transport, we will test serum samples diluted with phosphate buffer while still maintaining an appropriate linear range.

## Figures and Tables

**Table 1:** Glutathione sensitivity for three oxygen reduction enzyme electrodes.

Enzyme	Sensitivity (A/mM Glutathione)
Bilirubin oxidase	0.685
Laccase	1.044
Tyrosinase	0.039

## Supplementary Materials

The following are available online at https://www.mdpi.com/2079-6374/10/9/114/s1, Figure S1: Biosensor response at several glucose concentrations, Figure S2: Biosensor response in high chloride solution.
