# Supplementary material for: A Self-Powered Biosensor for the Detection of Glutathione
Source: Biosensors (Basel). 2020 Sep 3;10(9):114. doi: 10.3390/bios10090114 (PMC7558183; doi:10.3390/bios10090114)
Supplement: Supplementary file 1 [file biosensors-10-00114-s001.pdf]

biosensors

Supplementary materials

A Self-Powered Biosensor for the Detection of Glutathione

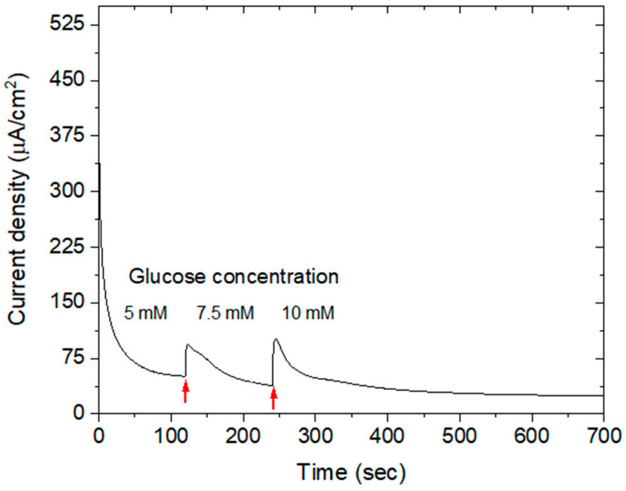

Figure S1. Current density measured at 0 V for a glucose biofuel cell in 0.1 M phosphate buffer, pH 7.4, with 100 µM glutathione and varying concentrations of glucose. Red arrows indicate the time glucose was added to the solution.

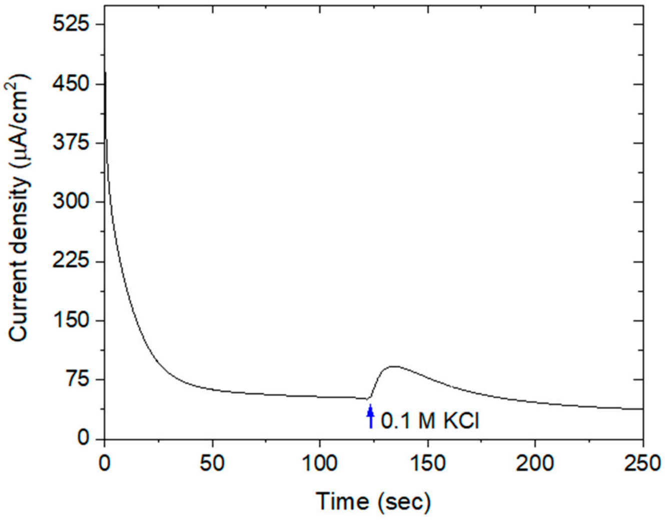

Figure S2. Current density measured at 0 V for a glucose biofuel cell in 0.1 M phosphate buffer, pH 7.4, with 100 µM glutathione and 10 mM glucose. The blue arrow indicates the time solution was added to a final concentration of 0.1 M KCl.
